# Design of the randomized, controlled sequential staged treatment of emphysema with upper lobe predominance (STEP-UP) study

**DOI:** 10.1186/1471-2466-14-190

**Published:** 2014-12-03

**Authors:** Arschang Valipour, Felix JF Herth, Ralf Eberhardt, Pallav L Shah, Avina Gupta, Robert Barry, Erik Henne, Sourish Bandyopadhyay, Greg Snell

**Affiliations:** Department of Respiratory and Critical Care Medicine, Ludwig-Boltzmann-Institute for COPD, Otto-Wagner-Hospital, Vienna, Austria; Department of Pneumology and Critical Care Medicine, Thoraxklinik, University of Heidelberg, Heidelberg, Germany; The National Institute for Health Research Respiratory Biomedical Research Unit, Royal Brompton, Harefield NHS Foundation Trust and Imperial College, London, UK; Imperial College London, London, UK; Chelsea & Westminster Hospital NHS Foundation Trust, London, UK; Uptake Medical Corp, Tustin, California USA; Department of Respiratory Medicine, Alfred Hospital and Monash University, Medical School, Melbourne, Australia

**Keywords:** Vapour ablation, STEP-UP, Emphysema, Lung volume reduction

## Abstract

**Background:**

An innovative approach to lung volume reduction (LVR) for emphysema is introduced in the design of the Sequential Segmental Treatment of Emphysema with Upper Lobe Predominance (STEP-UP) trial where vapour ablation is administered bilaterally over the course of two sessions and is used to target only the most diseased upper lobe segments. By dividing the procedure into two sessions, there is potential to increase the total volume treated per patient but reduce volume treated and energy delivered per session. This is expected to correlate with improvements in vapour ablation’s safety and efficacy profiles.

**Methods:**

The STEP-UP trial is a randomized, controlled, open-label, 12 month study of patients with upper lobe predominant emphysema (ULPE). The trial compares patients receiving standard medical management alone against patients receiving bilateral vapour ablation in addition to standard medical management. An intended sixty nine subjects will be randomized at a 2:1 (treatment arm:control arm) ratio. Inclusion criteria include a forced expiratory volume in 1 second (FEV_1_) between 20% and 45% predicted, total lung capacity > 100% predicted, residual volume > 150% predicted, marked dyspnea scoring ≥ 2 on the modified Medical Research Council (mMRC) scale, and PaCO2 ≤ 50 mm Hg. The primary endpoints are the change in FEV_1_ %predicted and St. George Respiratory Questionnaire (SGRQ) score between the treatment and control arm at 12 months. Adverse events will be monitored as secondary endpoints along with other efficacy outcomes at 6 and 12 months.

**Discussion:**

Vapour ablation can reduce lung volume in the presence of collateral ventilation (CV). Due to this ability, it can be used to target specifically the more diseased segments of each upper lobe. Safety and efficacy outcomes are expected to improve by considering which segments to treat along with the volume treated per session and per patient.

**Trial registration:**

ClinicalTrials.gov NCT01719263.

## Background

In 2004, the World Health Organization estimated that 64 million people have chronic obstructive pulmonary disease (COPD) [1]. Emphysema, a subset of COPD, damages the alveoli causing hyperinflation and reduced gas exchange resulting in a decrease in pulmonary function, shortness of breath and a decline in the patient’s quality of life. Although there is presently no cure for this progressive disease, data from the randomized National Emphysema Treatment Trial (NETT) identifies lung volume reduction surgery (LVRS) as an effective treatment for patients with emphysema [2]. Patients with upper lobe predominant emphysema (ULPE) and low exercise capacity had the best results with LVRS. By reducing the more diseased upper lobe, hyperinflation is reduced as measured by residual volume reduction and the better functioning lower lobes are preserved.

Although results from the NETT demonstrated an improvement in pulmonary function such as FEV_1_, exercise capability, and quality of life post-surgery, the NETT also demonstrated significant mortality and morbidity associated with surgery. Complications included prolonged hospital stays (25% >30 days), air leak (~90%), and respiratory or cardiac complications [2–4]. These complications have limited the adoption of LVRS as a routine therapy for patients with severe emphysema and have presented a medical need for an alternate effective method to achieve lung volume reduction (LVR) with an improved safety profile. To avoid the associated risk of surgery, various bronchoscopic techniques to induce LVR have emerged [5–8]. One bronchoscopic technique is vapour ablation (InterVapor, Uptake Medical, California, USA).

**Figure 1:**
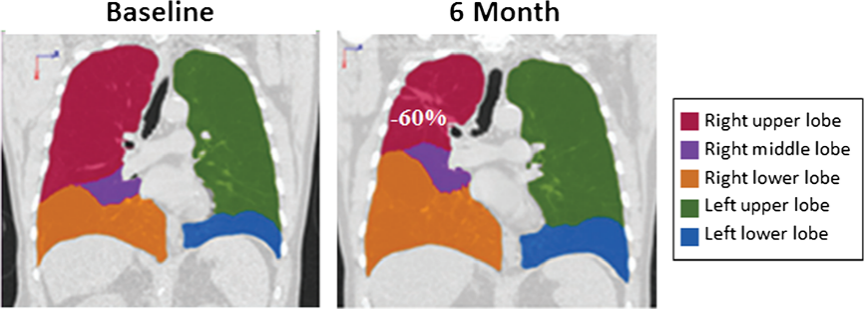
**Radiographic image of lungs pre- and post-vapor ablation treatment.** Radiographic image shows treatment of the right upper lobe resulting in targeted lung volume reduction with expansion of lower lobe following vapour ablation (VAPOR trial) [9].

Vapour ablation is a minimally invasive technique in which emphysematous lung regions are thermally ablated by bronchoscopic delivery of water vapour to targeted regions of lung [6, 9]. Vapour ablation is a bronchoscopic therapy that selectively targets emphysematous lung tissue resulting in a reduction of air and tissue volumes. The largest study to date for vapour ablation is the VAPOR trial. The VAPOR trial was designed for unilateral treatment of an entire lobe in 44 patients with ULPE and resulted in significant improvements in lung function, exercise capability, and health-related quality of life with acceptable morbidity [9, 10]. The trial proved vapour ablation successfully reduces lung volume (Figure 1) and results demonstrated that at 6 months post therapy, 83% of the subjects had clinically relevant improvement in either forced expiratory volume in 1 second (FEV_1)_ or St. George Respiratory Questionnaire (SGRQ) quality of life assessment. 55% of subjects had improvements in FEV_1_ of ≥ 12% and 73% of subjects had improvements in the SGRQ total score of ≥ 4 units at 6 months [9, 10].

**Figure 2:**
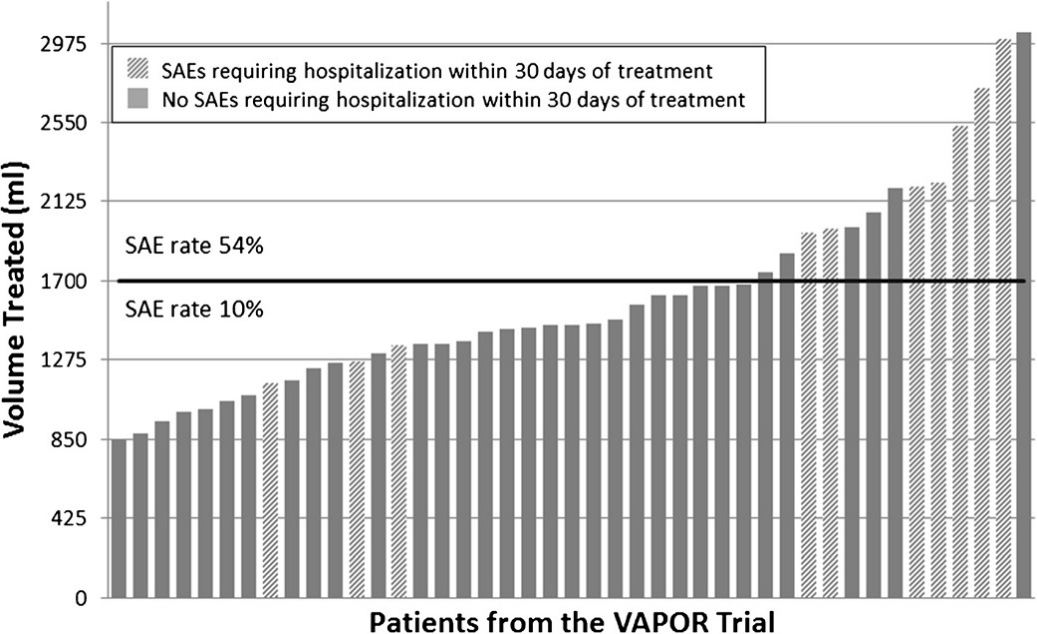
**Relationship between adverse event outcomes and volume treated.** Graphical representation of volume treated during the VAPOR trial to demonstrate the risk of having a SAE increases as the volume treated per session increases. Each patient is represented by a bar along the horizontal axis. Volume treated during the VAPOR trial is on the vertical axis. To promote safety, volume treated per session is now capped at 1700 ml. The SAE rate is 54% for treatments above 1700 ml and is much smaller at 10% for treatments larger than 1700 ml.

Post hoc analyses of the VAPOR trial cohort indicated that the occurrence of serious adverse events (SAEs) typically increases with the volume treated per session (Figure 2) [11]. Analysis also revealed an inflection point at 1700 ml treated above which the SAE rate was 54% and below which the SAE rate was 10% [11]. An innovative approach to further limit the volume treated per session is to treat certain segments individually rather than the entire lobe. To account for reducing less volume per session, the treatment can be administered bilaterally over the course of multiple sessions. It is hypothesized that treating smaller amounts of lung over the course of multiple sessions will lead to an improvement in the safety profile while maintaining or potentially improving efficacy. However, to effectively induce LVR at the segmental level (rather than at the lobar level), the treatment must be able to reduce volume irrespective of the presence of intralobar collateral ventilation (CV), a common feature of both normal and emphysematous lungs in humans [12, 13]. Vapour ablation has previously been shown to successfully induce LVR in the presence of CV and can therefore be used to target emphysematous tissue at the segmental level in addition to at the lobar level [14].

The purpose of this paper is to describe the design and rationale of the Sequential Segmental Treatment of Emphysema with Upper Lobe Predominance (STEP-UP) trial. This randomized, controlled trial uses vapour ablation in a stepped bilateral treatment to target individual segments based on disease state. The STEP-UP trial will investigate the approach of treating smaller amounts of lung over the course of two sessions to improve the safety profile while maintaining or potentially improving efficacy. Briefly, the STEP-UP trial’s algorithm targets one segment of an upper lobe during session 1 and steps up to treating up to two segments of the contralateral lobe during session 2. The details of the STEP-UP trial’s algorithm to identify the most diseased segments are further described in the Methods section. This sequential segmental approach reduces the overall energy delivered and the volume of lung treated in a single session while increasing the volume of lung treated during the entire procedure in comparison to VAPOR. Consequently, the reduced application of energy per session is expected to reduce the occurrence of SAEs associated with vapour ablation and improve the safety profile while the overall increase in volume of lung treated per procedure is expected to improve the efficacy profile. Furthermore, studies have indicated that bilateral treatments, which typically treat more volume than unilateral treatments, may result in greater efficacy for pulmonary function measures, such as FEV_1_ [15, 16]. For this reason, the STEP-UP algorithm will treat bilaterally in a stepped manner and typically target a larger volume of emphysematous lung per patient over the course of two sessions in comparison to the VAPOR trial. Because not all upper lobe segments are being treated, it is possible to target and reduce the most diseased segments of each upper lobe allowing the untreated portions of less diseased tissue to expand. This concept has been proven to yield the best clinical benefit for the patient and the STEP-UP trial aims to prove that the concept also holds true on a segmental basis [17–19]. These goals are outlined in Table 1.

**Table 1:** **Goals for sequential segmental treatment**

Step- up goals	Reasoning
Treat less lung volume per session as compared to the lobar treatment sessions in VAPOR	The rate of SAEs increases as volume of lung treated increases [11]. Reducing volume per session is expected to reduce the rate of SAEs.
Treat a larger amount of lung volume per patient as compared to patients in VAPOR	Change in FEV_1_ is related to the amount of diseased lung tissue treated per patient. Increasing the volume of diseased lung to be treated per patient is expected to result in an improvement of pulmonary function and efficacy [16–18].
Treat the most diseased segments in the upper lobes	The NETT demonstrates that reducing the more diseased upper lobes allow healthier lower lobes to expand yielding the best benefit. It is therefore hypothesized that targeting more diseased portions (segments) of the upper lobe should allow the least diseased regions of the upper lobes, in addition to the lower lobes, to expand [17, 19].
Preserve least diseased segments of the upper lobes	Emphysematous tissue is typically not homogeneous (equivalently diseased) throughout the lung or lobe. Preserving the least diseased segments allowing them to expand is expected to improve gas exchange in the upper lobes.

## Methods/Design

### Trial design

The STEP-UP trial is a randomized, controlled, open-label, 12 month study of patients with upper lobe predominant emphysema. 69 subjects are intended to be randomized at a 2:1 (treatment arm: control arm) ratio using a blinded blocked randomization scheme separated by site. The trial will compare patients receiving standard medical management alone against patients receiving bilateral vapour ablation treatment in addition to standard medical management. Medical management will be consistent with Global Initiative for Chronic Obstructive Lung Disease (GOLD) guidelines and will consist of a progressive dosage of one or more bronchodilators, inhaled corticosteroids, and oxygen therapy. Up to 17 hospital sites will participate in the study.

### Screening assessments and HRCT

A completed patient informed consent form is required from all patients participating in the study. The form is reviewed and approved by the Institutional Review Board or Ethics Committee of all participating hospital sites. The informed consent forms are developed in accordance to the current guidelines outlined by Good Clinical Practice guidelines, the Declaration of Helsinki, and the International Conference on Harmonisation.

All patients will undergo two screening phases. The initial screening is completed within 30 days of randomization and assesses patient eligibility as defined by the inclusion and exclusion criteria. Because the NETT and STEP-UP trial both have similar goals (i.e. achieve LVR), the NETT has been used to establish many of the vapour ablation treatment criteria. Additional criteria have also been established using results from the VAPOR trial. The STEP-UP trial inclusion and exclusion criteria are outlined in Tables 2 and 3. Detailed STEP-UP trial inclusion/ exclusion criteria can be found at http://clinicaltrials.gov/show/NCT01719263.

**Table 2:** **The STEP-UP trial inclusion criteria**

1.	Age ≥ 40 and ≤ 75 years old
2.	Heterogeneous emphysema with upper lobe predominance in both lungs
3.	FEV1 between 20% and 45% predicted
4.	Total lung capacity (TLC) ≥ 100% predicted
5.	Residual volume (RV) > 150% predicted
6.	Post-rehabilitation 6-minute walk test > 140 meters
7.	Marked dyspnea scoring > 2 on the modified Medical Research Council scale (mMRC)
8.	Arterial blood gas levels of: PaCO2 ≤ 50 mm Hg; PaO2 > 50 mm Hg on room air
9.	Non-smoking for 6 months prior to study enrollment
10.	Optimized medical management (treatment consistent with GOLD guidelines)
11.	Evidence of completed pulmonary rehabilitation:
	a) ≥ 6 weeks out-patient or ≥ 3 weeks in-patient within 6 months of enrollment; or,
	b) Patient has or continues to participate in regular physical activity beyond activities of daily living (i.e. a walking program) for ≥ 6 weeks under the supervision of a health care professional
12.	Mentally and physically able to cooperate with the study procedures and to provide informed consent to participate in the study.

**Table 3:** **The STEP-UP trial exclusion criteria**

1.	Any condition that would interfere with the completion of the study follow-up assessments, bronchoscopy, or that would adversely affect study outcome.
2.	FEV1 < 20% predicted
3.	DLCO < 20% predicted
4.	Body mass index (BMI) < 18 kg/m2 or > 32 kg/m2
5.	Pulmonary hypertension:
	a) Peak systolic PAP > 45 mm Hg or Mean PAP > 35 mm Hg
	b) Right heart catheter measurements will be considered definitive over echocardiogram measurements
6.	Inability to walk > 140 meters in 6 minutes (6MWD) following optimized medical management and prescribed rehabilitation
7.	Homogeneous disease and/or with highly diseased lower lobes (Density - tissue to air ratio of <11%)
8.	Clinical significant bronchiectasis
9.	Pneumothorax or pleural effusions within previous 6 months
10.	Heart and/or lung conditions, stroke, heart failure, transplant, lung volume reduction or resection, bullectomy, or implantable cardiac defibrillator implant
11.	Recent COPD exacerbation in preceding 6 weeks, or > 3 COPD related hospitalizations requiring antibiotics in past 12 months
12.	Daily use of systemic steroids, > 5 mg prednisolone
13.	Single large bulla (defined as > 1/3 volume of the lobe) in upper lobe
14.	Coagulopathy or current use of anticoagulants

During the initial screening visit, patient demographics, smoking history, physical examination, pulmonary and concomitant medication use, and medical history is recorded. Additional screening also includes completing SGRQ, Modified Medical Research Council Dyspnea Score, COPD Assessment Test, post-bronchodilation body plethysmography, post-bronchodilation diffusing capacity of the lungs for carbon monoxide (DLCO) test, 12-lead electrocardiography, arterial blood gas, six minute walk test, and a high resolution computed tomography (HRCT) scan. The HRCT scan will be used for procedure planning, assessing inclusion criteria including heterogeneity of the disease and lower lobe TAR, and generating treatment plans for both treatment sessions.

The second screening visit is completed within 7 days of randomization to define baseline data closer to the randomization date and to ensure the patient still qualifies. This visit will consist of a physical examination including vital signs, post-bronchodilation spirometry, a review of the subject’s daily diary, concomitant medications, adverse events, and exercise counselling. A complete list of screening assessments is available at http://clinicaltrials.gov/show/NCT01719263.

#### Assessing heterogeneity for inclusion criteria

A patient HRCT will be acquired within 90 days of randomization and will be used to assess patient eligibility, such as heterogeneity, and create patient treatment plans for the first and second treatments. The process for determining heterogeneity in the STEP-UP trial is more quantifiable than the process used during the NETT, where radiologists were assigned the task of qualitatively determining heterogeneity. During the NETT trial, each lung was divided into 3 zones and each zone was ranked on a scale of 0–4 to represent the severity of emphysema where 0 indicated no emphysema and 4 indicated a high severity of emphysema. Lungs with a difference in ranking of at least 2 in two of the three zones were considered heterogeneous [20]. This method, however, is subjective and does not necessarily follow natural anatomical boundaries, such as fissures. In the STEP-UP trial, quantitative CT analysis is used to assess heterogeneity precisely along anatomical boundaries. Tissue and air values are calculated for each segment using quantitative CT analysis. The left upper lobe is comprised of 5 segments: LB1, LB2, LB3, LB4, and LB5. LB4 and LB5 together form the lingula, which is not considered for upper lobe predominant treatment using vapour ablation. The right upper lobe is comprised of 3 segments: RB1, RB2, and RB3, all of which are considered for upper lobe predominant treatment using vapour ablation. The tissue and air values are used to evaluate the tissue to air ratio (TAR), a measure of lung density and disease state, for each segment and lobe. TAR is then used to calculate the heterogeneity index (HI), a ratio that evaluates the disease of the upper lobe relative to the lower lobe. Because the STEP-UP trial targets treatment of individual segments rather than an entire lobe, the HI is calculated at a segmental level using segmental TAR (Eq. 1). The STEP-UP heterogeneity inclusion criterion requires all segments treated to have a baseline HI of no less than 1.2.
1

#### Assessing lower lobe TAR for inclusion criteria

Quantitative CT analysis is also used to evaluate lower lobe TAR. A STEP-UP exclusion criterion is a lower lobe TAR of less than 11%. Analyses of a small number of previous vapour ablation cases (not shown) suggest patients with a baseline lower lobe TAR < 11% may have a reduced safety profile in comparison to patients with a baseline lower lobe TAR ≥ 11%.

### Treatment algorithm

**Figure 3:**
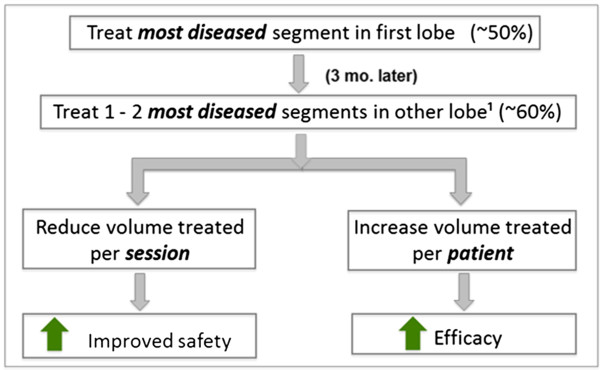
**Hypothesized improvements in safety and efficacy using the STEP-UP algorithm.** The STEP-UP trial’s treatment volume parameters lead to a hypothesized improvement in safety and efficacy.

To pursue a balance of safety and efficacy, the following treatment volume parameters have been set (Figure 3):

- Treat one preferentially most diseased segment meeting segmental HI criteria during session 1.
- Treat up to two preferentially most diseased segments meeting segmental HI criteria during session 2.
- Treatment session 1 will target treatment of 50% (±20%) of the upper lobe (referred to as the primary lobe).
- Treatment session 2 will target 60% (±20%) of the contralateral upper lobe (referred to as the secondary lobe).
- Combination of both sessions will target treatment of 110%* (+20%/-15%) of the two lobes treated.
- Volume treated per session to not exceed 1700 ml.

*Due to the anatomical tendency of the two upper lobes to differ in size, the percentages treated per patient cannot be combined to be evaluated out of 100%. Instead, one entire lobe is evaluated as 100% and the combination of both lobes is evaluated as 200%.

The STEP-UP trial targets the most diseased segments as defined by TAR (density). Because hyperinflation of the lung causes air pockets to develop during emphysema, density of the segment decreases as it becomes more diseased [21]. Similarly, because TAR is a measure of lung density, lower TAR is associated with the more diseased segment. To consistently apply all criteria for segment selection to each patient, a matrix of all possible treatment options using the 3 available segments in each upper lobe (left: LB1, LB2, LB3; right: RB1, RB2, RB3) is generated and used for each patient. Note that LB1 and LB2 can be combined to form LB1 + 2 and be treated as one segment.

How these segments are selected to optimally meet these parameters is as follows. All possible treatment options are generated and considered where each treatment option is treatment of 1 segment from the primary lobe and up to two segments of the secondary lobe. Each treatment option is evaluated to determine whether each session and overall procedure meets the treatment volume parameters and whether each segment meets HI criteria (≥1.2). Any treatment options that do not meet these criteria are removed. Next, the distribution of emphysema disease within each upper lobe is evaluated.

Each segment is evaluated to determine which one is the least diseased within a lobe. Segmental TAR is calculated for each segment to determine severity of disease using quantitative CT analysis. Segments within a lobe that have a segmental TAR within 2% of each other are considered equally diseased. Otherwise, the segment with the smallest segmental TAR is considered to be most diseased and the segment with the largest segmental TAR is considered to be least diseased. Any treatment option proposing treatment of the least diseased segment in an upper lobe is removed from consideration unless all remaining treatment options treat such a segment. If only one treatment options remains, it will be used as the preferred treatment plan. Otherwise, the combined TAR of the upper lobe segments will be used to determine the preferred treatment option (Eq. 2).
2

Combined TAR compiles the segmental TAR associated with each segment in a treatment option to determine which one targets the most disease. The treatment option with the lowest combined TAR treats the most disease and is therefore chosen to pursue a treatment plan. However, any treatment options within 0.30% combined TAR are considered to be treating similar amounts of disease. If two or more treatment options are considered to be treating a similar amount of disease, the treatment option that is closer to treating the target volume of 110% of the patient’s upper lobes will be used to pursue the patient’s preferred personalized treatment plan.

### Primary and secondary endpoints

The STEP-UP primary endpoints are the change in FEV_1_ (% predicted) and SGRQ score between the treatment and control arm at 12 months. Adverse events will be monitored over the course of the study and be considered as secondary endpoints along with other efficacy outcomes at 6 and 12 months such as lung volumes, mMRC, SGRQ, DLCO, 6MWT. LVR from a 6 month follow-up HRCT will also be a secondary efficacy endpoint for the treatment group.

### Patient schedule and follow up

Patients will be randomized at a 2:1 ratio to the treatment arm. These patients will receive standard medical management and vapour ablation treatment over the course of two sessions. The first treatment session will take place within 7 days of randomization and will be denoted as study day 1. The second treatment session will be scheduled 13 weeks after the first treatment session (+/- 7 days). However, the treating physician may delay the second treatment session by up to 4 weeks if the chest x-ray or inflammatory markers indicate the healing process has not completed prior to the second treatment session. After each treatment session, patients should continue prophylactic broad spectrum antibiotics ≥ 14 days, remain active, and be encouraged to incorporate regular physical activity. If moderate to severe symptoms occur, patients should be informed to contact their treating physician. Moderate to severe symptoms are treated with increased bronchodilators, antibiotics and oral steroids. One third of the patients will be randomized to the control arm and will receive standard medical management alone without vapour ablation treatment. The date of randomization is denoted as study day 1 for control arm patients.

**Figure 4:**
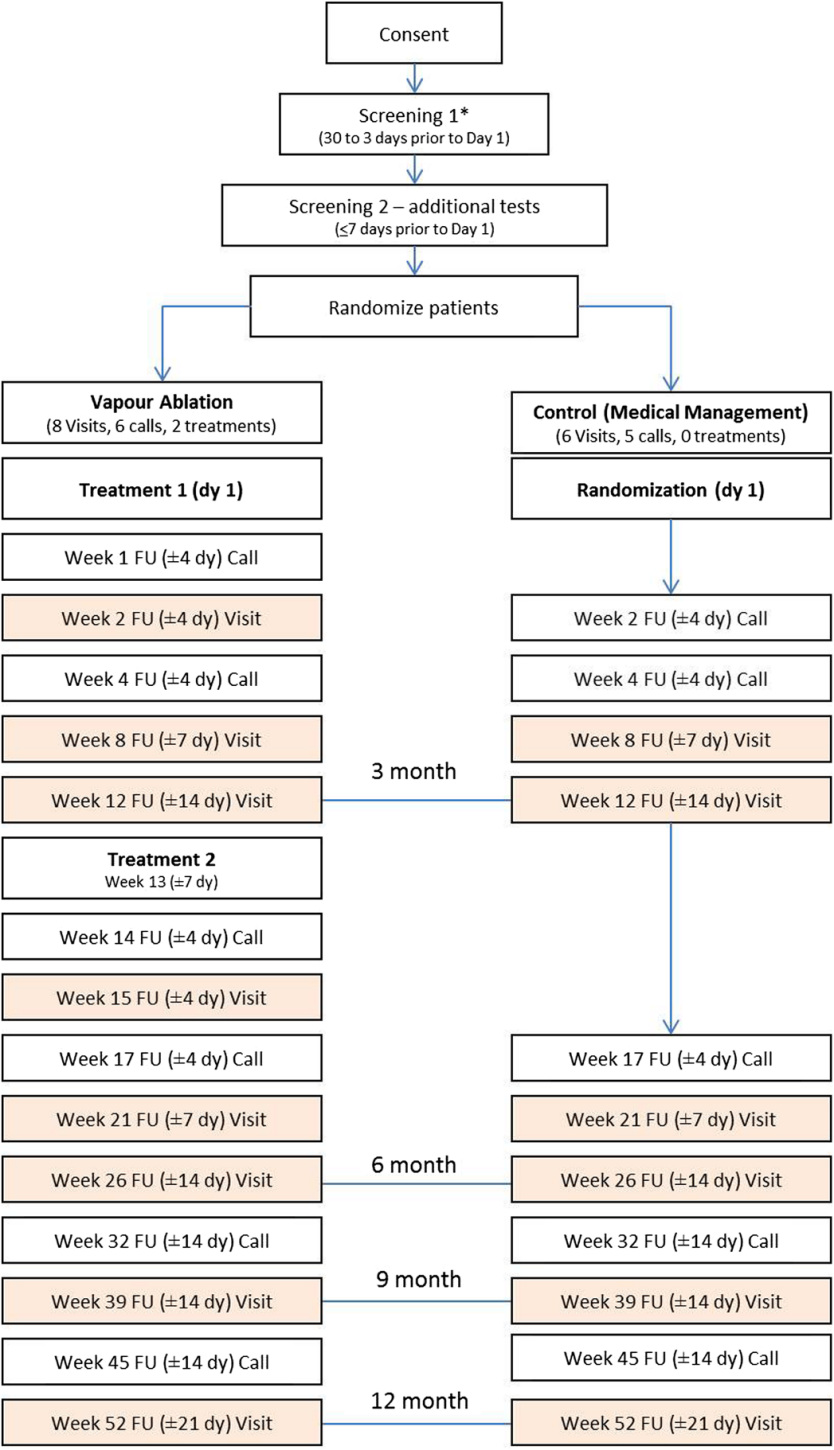
**Patient follow-up visit schedule.** *The HRCT scanned for the screening process can be acquired up to 90 days before randomization. FU = follow-up.

Patient follow-up visits will be scheduled as telephone calls and in-person clinic visits. During telephone calls, research coordinators or trained designees will review the daily diary, record any change in medications, any occurrence of adverse events, and review exercise counselling. The clinic visits will consist of a physical exam, review of vital signs, review of medication, and review of any adverse events that may have occurred since the previous follow-up visit or telephone call. If a COPD exacerbation occurs during the conduct of the clinical trial, treatment of the exacerbation should follow standard medical care and may include the addition of, or increase in systemic corticosteroids (generally prescribed for up to 2 weeks) and/or antibiotics. In the case of severe exacerbations, any and all therapies and interventions deemed medically necessary by the treating physician may be prescribed.Telephone calls will be scheduled at 1, 4, 14, 17, 32, and 45 weeks and in-person clinic visits will be scheduled at 2, 8, 12, 15, 21, 26, 39 and 52 weeks (Figure 4). Control patients will not be scheduled for the follow-up visits at weeks 1, 14 and 15. Also, week 2 will be scheduled as telephone calls rather than clinic visits for patients randomized to the control arm. A HRCT will also be acquired at the 6 month timeline for the treatment arm only, which will be used to assess LVR post-treatment.

### Analysis

A two-sample t test was used to determine the sample size for the study based on the two primary endpoints. An assumption of a 12% predicted difference in FEV_1_ between the treatment arm and the control arm at 12 months with an assumed standard deviation of 15 leads to an intended sample size of 59 patients. An assumption of an 11 point difference in SGRQ between the treatment arm and the control arm at 12 months with a standard deviation of 14 leads to an intended sample size of 60 patients. However, to account for an estimated 15% loss during patient follow-up, the sample size was increased by 15% resulting in an intended sample size of 69 patients. The sample size was calculated using an 80% power, a Type I error rate of 0.05 and a 2:1 randomization allocation. The calculation was computed using Power Analysis and Sample Size 11 software.

The Hochberg method will be utilized to compensate for the multiple comparisons resulting from two endpoints and evaluate if the primary endpoint has been reached. The Hochberg method allows for two different scenarios to determine achievement of the primary endpoint for a positive trial:

1. If both primary endpoints (FEV_1_ %predicted and SGRQ) reach clinical significance at the 0.05 level.

OR

2. If either one of the two endpoints (FEV_1_ %predicted or SGRQ) reach clinical significance at the 0.025 level.

Additionally, a binary responder rate analysis will be performed, where a responder is identified for the following secondary endpoints as:

- %predicted FEV_1_ ≥ 12% difference from baseline
- SGRQ ≥ 8 points difference from baseline
- 6MWT ≥ 30 meters from baseline

Primary endpoint analysis will be based on the 12 month visit. If data is not available due to a serious adverse event, including death, then the worst prior follow-up value will be used. If data is missing in other cases, the last available value from prior visits will be used. If no follow-up data is available, an intent-to-treat analysis will use the baseline values. A sensitivity analysis will be presented excluding such patients.

### Safety

A Data Safety Monitoring Board (DSMB) and Clinical Events Committee (CEC) are in place to closely and independently monitor the safety of the STEP-UP trial. Reports outlining these occurrences will be generated on a frequent periodic basis unless the DSMB/CEC requests the reports more often. DSMB/CEC will conduct a review at a minimum for the first and second treatment of the first 15, 30 and 46 patients.

## Discussion

The NETT established that although LVRS may lead to clinically relevant improvement for patients with ULPE, the treatment is also associated with a high risk profile. To minimize these risks, LVR can be induced bronchoscopically via vapour ablation. Pilot studies for vapour ablation, including the VAPOR trial, have demonstrated successful LVR and clinically significant improvement for patients treated unilaterally with vapour ablation [5, 10]. The STEP-UP trial has been established to investigate a bilateral sequential segmental approach for vapour ablation and has been designed to conserve a balance between the treatment’s safety and efficacy profiles.

Vapour ablation treatment ablates emphysematous tissue resulting in a reduction of lung volume and hyperinflation. Each STEP-UP procedure will consist of two treatment sessions and each session will treat smaller volumes of lung in comparison to VAPOR. This is expected to reduce the occurrence of SAEs during the STEP-UP trial due to correlation defined previously between amount of lung treated per session and rate of SAEs. In addition to reducing the amount of energy delivered per session, treating over the course of two sessions typically allows a larger volume of lung to be treated during the entire procedure in comparison to the VAPOR trial. The increase in total volume treated per procedure is anticipated to improve efficacy while lessening the amount of energy delivered in one session is anticipated to improve safety.

The severity of disease of each segment targeted for treatment is also anticipated to affect the improvement in efficacy. Due to vapour ablation’s ability to treat in the presence of CV, the treatment can specify and target individual segments. The STEP-UP trial explores the approach of targeting only the most diseased segments for treatment of both upper lobes to allow the less diseased segments to continue lung functionality.

Furthermore, studies have demonstrated that the larger the heterogeneity, the more efficacious the outcomes are post-LVRS [22, 23]. This has also held true for LVR induced via treatment using an alternate bronchoscopic method (endobronchial valves), where larger heterogeneity showed correlation with improvements in FEV_1_ and 6MWT [24]. This indicates that LVR is most beneficial when reducing the most diseased portions of the lung. Because LVR has traditionally been induced at the lobar level, heterogeneity has also traditionally been defined as the difference in disease state between ipsilateral lobes. However, because the STEP-UP trial targets individual segments for treatment, it is important to apply the idea of heterogeneity at the segmental level. Segments within a lobe are not necessarily equivalently diseased and applying heterogeneity at the segmental level determines a segment’s disease state in comparison to the remaining lobe as well as disease state in comparison to the ipsilateral lower lobe. This helps in identifying the segment that is least contributing to healthy pulmonary function or conversely most contributing to poor pulmonary function. The segments that are relatively less diseased in comparison to the remaining emphysematous upper lobe and lung positively contribute to pulmonary function and should therefore be conserved.

If we look to the future potential of bronchoscopic LVR in general and vapour therapy in particular we note that 1) due to the progressive nature of emphysema, the segments are expected to worsen overtime causing pulmonary function to decline, 2) because not all segments are treated during the STEP-UP trial, the segmental approach may potentially also be utilized in the future as a follow-up treatment for patients who have previously been treated for emphysema and 3) a focused segmental approach across all affected lobes might elucidate therapeutic targets in whole patient populations previously generalized as having a diffuse disease.

### Study limitations

One limitation of the study is the difficulty to fully blind the trial. A fully blinded approach accentuates the scientific merit of the study results and was therefore considered, but was deemed not appropriate for this study. A single or double blinded approach would require control patients to undergo two false bronchoscopy sessions over the course of the study while under local anesthesia. This would present unnecessary and ethically questionable risk to the patients and was therefore decided to not be appropriate for this study. For this reason, the investigator and patient are aware of the treatment status. However, the investigators, patients, and sponsors, are blinded from the summary statistics during the treatment phase of the study.

## Conclusion

The effects of emphysema are dependent on the heterogeneity and severity of the disease. Significantly affected regions are depicted by low density areas on HRCT and have greater parenchymal destruction resulting in hyperinflation and reduced pulmonary function. For this reason, it is hypothesized that the ability to specifically reduce the most emphysematous segments will yield the most effective results. Previously, the NETT trial visually assessed heterogeneity to determine upper and lower lobe predominance. Results demonstrated that the more diseased the reduced lung region and the less diseased the remaining lung region, the greater the clinical improvement for the patient. Similarly, the VAPOR trial also assessed heterogeneity of the lung to typically reduce an entire lobe during one session using vapour ablation resulting in clinical benefit. The STEP-UP trial is able to take this proven concept of effective volume reduction from the lobar level of the NETT and VAPOR studies and apply it to the segmental level by virtue of the fact that vapour ablation uniquely achieves volume reduction in the presence of CV. A potential advantage of the STEP-UP trial is an improved safety profile over the NETT and VAPOR studies. The STEP-UP trial’s algorithm treats relatively small amounts of volume overtime leading to delivery of relatively small amounts of energy to a patient during each session. Additionally, there is potential for improved efficacy by treating only the most diseased segments and thereby leaving less diseased segments intact to contribute to pulmonary function post-procedure. These segments will be treated over the course of two sessions to allow larger diseased portions of the lung to be treated per patient without increasing the energy delivered per session. The STEP-UP trial results will elucidate whether the hypothesis and associated benefits presented in this paper hold true.

## Abbreviations

COPD: Chronic obstructive pulmonary disease
CV: Collateral ventilation
DLCO: Diffusing capacity of the lungs for carbon monoxide
FEV_1_: Forced expiratory volume in 1 second
HI: Heterogeneity index
HRCT: High resolution computed tomography
LVR: Lung volume reduction
LVRS: Lung volume reduction surgery
NETT: National emphysema treatment trial
SAE: Serious adverse events
SGRQ: St. George respiratory questionnaire
STEP-UP: Sequential segmental treatment of emphysema with upper lobe predominance
TAR: Tissue to air ratio
ULPE: Upper lobe predominant emphysema
6MWT: Six minute walk test.
